# Parasite-Microbiota Interactions With the Vertebrate Gut: Synthesis Through an Ecological Lens

**DOI:** 10.3389/fmicb.2018.00843

**Published:** 2018-05-14

**Authors:** Jacqueline M. Leung, Andrea L. Graham, Sarah C. L. Knowles

**Affiliations:** ^1^Department of Ecology and Evolutionary Biology, Princeton University, Princeton, NJ, United States; ^2^Royal Veterinary College, Hatfield, United Kingdom

**Keywords:** parasite, gut microbiota, helminth, protozoa, interactions, probiotic, germ-free, gnotobiotic

## Abstract

The vertebrate gut teems with a large, diverse, and dynamic bacterial community that has pervasive effects on gut physiology, metabolism, and immunity. Under natural conditions, these microbes share their habitat with a similarly dynamic community of eukaryotes (helminths, protozoa, and fungi), many of which are well-known parasites. Both parasites and the prokaryotic microbiota can dramatically alter the physical and immune landscape of the gut, creating ample opportunities for them to interact. Such interactions may critically alter infection outcomes and affect overall host health and disease. For instance, parasite infection can change how a host interacts with its bacterial flora, either driving or protecting against dysbiosis and inflammatory disease. Conversely, the microbiota can alter a parasite's colonization success, replication, and virulence, shifting it along the parasitism-mutualism spectrum. The mechanisms and consequences of these interactions are just starting to be elucidated in an emergent transdisciplinary area at the boundary of microbiology and parasitology. However, heterogeneity in experimental designs, host and parasite species, and a largely phenomenological and taxonomic approach to synthesizing the literature have meant that common themes across studies remain elusive. Here, we use an ecological perspective to review the literature on interactions between the prokaryotic microbiota and eukaryotic parasites in the vertebrate gut. Using knowledge about parasite biology and ecology, we discuss mechanisms by which they may interact with gut microbes, the consequences of such interactions for host health, and how understanding parasite-microbiota interactions may lead to novel approaches in disease control.

## A transdomain ménage à trois

Prokaryotes and parasitic eukaryotes have cohabited the vertebrate intestinal tract for hundreds of millions of years, over which time the immune system itself has evolved (Jackson et al., 2009). During this time, biotic interactions among these two groups and the host are expected to have driven co-evolution and shaped phenotypes in all three parties. A growing body of literature is starting to reveal how gut-dwelling eukaryotic parasites and the gut microbiota (here defined as the community of prokaryotes) may interact in vertebrates. For both microbiologists and parasitologists, understanding these interactions may be transformative for tackling major outstanding questions in these traditionally taxonomically focused fields. For example, both gastrointestinal helminths and members of the microbiota have been separately credited for their immunomodulatory abilities and contribution to immune homeostasis within hosts (McSorley et al., 2013; Honda and Littman, 2016). However, recent work suggests that interactions between them may also be an important piece of this puzzle, together shaping the evolution of the host immune system (Giacomin et al., 2015; Gause and Maizels, 2016). Transdomain interactions in the gut may also help explain the great variability and context-dependency of gut symbiont pathogenicity. Many, if not most, gut-dwelling organisms move along the parasitism-mutualism spectrum in a context-dependent manner (Méthot and Alizon, 2014). For example, parasitic protozoa in the genera *Toxoplasma, Giardia*, and *Entamoeba* only cause disease in a subset of cases, with many carriers remaining asymptomatic (Parfrey et al., 2011), and helminths, while detrimental at high burdens, can also be mutualistic in some contexts (Wammes et al., 2014). Similarly, many gut microbes can be considered pathobionts (Box 1), in that they do not ordinarily cause harm, but are capable of causing disease in certain contexts. Studies are now beginning to show that interactions between gut microbes and parasites can alter each other's pathogenicity (Box 1), suggesting that the community context in which these organisms survive is an important factor explaining variable virulence (Box 1).

Box 1 **Glossary**

**Colonization resistance:** Phenomenon by which commensal bacteria protect host intestines from exogenous pathogens.

**Community perturbation experiment:** Selective alteration of the density of one or more members of a community to observe changes in a secondary variable of interest.

**Cross-feeding or syntropy:** A relationship in which one organism consumes metabolites produced by another.

**Germ-free:** Conditions in which animals are reared and maintained in an environment such that there are no microorganisms living in or on them.

**IL-10:** Anti-inflammatory cytokine that limits T cell activation and suppresses pro-inflammatory responses in tissues.

**IL-17A:** Pro-inflammatory cytokine involved in host defenses against extracellular pathogens through the induction of neutrophils and macrophages to inflammatory sites.

**IL-22:** Cytokine involved in regulating intestinal inflammatory responses through the induction of antimicrobial peptides and the enhancement of epithelial regeneration and wound repair.

**Pathobiont:** A symbiont that is normally innocuous to hosts, but under certain conditions, has the potential to cause dysregulated inflammation and lead to disease.

**Pathogenicity:** A qualitative trait referring to the ability of a microorganism to harm a host and cause disease.

**Prebiotic:** Dietary substrates that stimulate the growth or activities of specific gut microbes in order to confer a health benefit to a host.

**Probiotic:** Live microorganisms that can provide a health benefit on a host when administered in adequate amounts.

**TGF-β:** Cytokine involved in the induction of peripheral tolerance.

**Short-chain fatty acids:** End products of bacterial fermentation that can regulate systemic immune responses through the induction of regulatory T cells.

**Virulence:** A quantitative trait referring to the degree of pathology caused by a microorganism.

## Parasite-microbiota interactions viewed through an ecological lens

Studies reporting effects of parasites on the microbiota or vice versa are becoming increasingly common, aided in recent years by improved access to next-generation sequencing technology. However, findings often vary widely across studies, no doubt partly due to variation in the experimental design, animal housing, and techniques used (Peachey et al., 2017). For example, considering the effect of helminth infections on the microbiota, parasite species do not seem to strongly predict how bacterial community composition or diversity will change upon infection, as study findings can be variable even for single, host-parasite systems. In Supplementary Table S1, we describe predominant changes to the gut microbiota with different helminth species within hosts. Controlled infection studies using the nematode *Trichuris muris*, for example, report somewhat variable findings regarding the bacterial taxa that change with infection, despite using similar infective doses, sampling time points, and sample types (Holm et al., 2015; Houlden et al., 2015; Supplementary Table S1). While challenging, understanding the mechanisms by which gut parasites and microbes affect one another may help to make sense of variation among studies, reveal predictors of context-dependent effects, and provide deeper insight into the consequences of such interactions for host health. Therefore, instead of a taxonomic or phenomenological approach, we review the growing literature on this topic using an ecological and mechanistic perspective. First, by considering the ecology of particular parasites and microbes within the gut (what they do and how they may alter the ecosystem), we predict key mechanisms of parasite-microbiota interactions and document the evidence they occur. We then discuss the potential consequences of such interactions for host health and disease, consider how knowledge about such interactions may lead to improvements in disease management, and finally highlight key open questions in this field. Although the gut eukaryome is diverse (Lukeš et al., 2015), our focus is on parasitic protozoa and helminths since these have received the most attention thus far, and many important interactions involve processes associated with virulence such as mucosa invasion or a defensive immune response. Little research has focused on gut-dwelling fungi (Gouba and Drancourt, 2015) or the many protozoa with unknown or low pathogenicity (but see Chudnovskiy et al., 2016). This is an area of study poised for future developments.

## Mechanisms of gut parasite-microbiota interaction

### Changes to the physical gastrointestinal landscape: mucus and the epithelial barrier

Colonization of the gastrointestinal (GI) tract by a parasite markedly alters physical aspects of the gut ecosystem and hence the landscape in which the microbiota reside. Infection can alter epithelial barrier function by affecting mucus production and composition, tight junctions, as well as epithelial cell turnover. Since the outer mucus layer houses and feeds many gut microbial taxa, and barrier function mediates access to and interaction with host immune cells, changes at the epithelial interface represent a key arena for parasite-microbiota interactions (Table 1).

**Table 1:** Mechanisms of parasite–microbiota interactions in the vertebrate gut.

**Mechanism category**	**Host factors involved**	**Effect direction**	**Mechanism description**	**(Potential) Consequences**	**Examples showing both mechanism and consequence**
**INDIRECT INTERACTIONS (INVOLVING THE HOST)**					
Physical changes to the gut	Intestinal mucus	P > M	Helminths, and some protozoa, increase mucus production	Increases mucolytic bacteria and bacteria capable of using mucins as a carbon source	*T. suis* (Li et al., 2012); *T. muris* (Holm et al., 2015; Houlden et al., 2015; Ramanan et al., 2016); *Eimeria* (Collier et al., 2008)
				Reduces bacteria attachment to the gut epithelium	*T. trichiura* (Broadhurst et al., 2012)
			Parasites alter mucus composition and structure	Alters food availability, attachment sites, gut flow rates, and access to the epithelium for gut microbes	*T. muris* (Hasnain et al., 2011); *N. brasiliensis* (Tsubokawa et al., 2015); *E. histolytica* (Hicks et al., 2000); *T. gondii* (Kim and Khan, 2013; Trevizan et al., 2016); *Giardia* (Kim and Khan, 2013)
		M > P	Microbiota affects mucus synthesis	Impacts expulsion rate of parasites	
	Epithelial barrier	P > M	Parasites damage epithelial tight junctions	Allows for microbial translocation across the gut epithelium	*H. polygyrus* (Chen et al., 2005); *T. spiralis* (McDermott et al., 2003); *S. venezuelensis* (Farid et al., 2007); *N. brasiliensis* (Hyoh et al., 1999); *T. gondii* (Heimesaat et al., 2006; Hand et al., 2012; Cohen and Denkers, 2014); *Giardia* (Chen et al., 2013; Halliez, 2014)
		M > P	Microbiota strengthens and shapes permeability of mucus barrier	Alters the degree of mucosal damage and bacterial translocation that occurs after parasite infection	
	Epithelial cell turnover	P > M	Helminths increase epithelial cell turnover	Selects for microbes capable of replicating at a high rate	
		M > P	Microbiota mediate cell turnover via SCFAs	Impacts parasite colonization and expulsion	
Innate immunity	Toll-like receptors	P > M	Helminths increase expression of TLRs	Increases activation of responses against microbiota	*H. polygyrus* (Ince et al., 2006; Friberg et al., 2013); *H. diminuta* (Kosik-Bogacka et al., 2012)
		M > P	Microbiota can prime protective immune responses through TLRs	Protects against parasite infection through primed innate immune responses	*T. gondii* (Benson et al., 2009)
	Antimicrobial peptides	P > M	Helminths secrete antimicrobial peptides	Protects against harmful immune responses elicited by microbial contact	
	Inflammasomes	P > M	Parasites alter inflammasome activation	Alters pro-inflammatory cytokine secretion and microbial dysbiosis	*T. musculis* (Chudnovskiy et al., 2016)
		M > P	Microbiota-derived metabolites activate inflammasomes	Creates a pro-inflammatory environment that may aid protozoa clearance, but also increased helminth chronicity	
Adaptive immunity	Th2 cells	P > M	Helminths increase Th2 responses	Alters mucosal barrier function and impairs TH1 responses leading to an inability to control bacterial replication	*H. polygyrus* (Chen et al., 2005)
		M > P	Gut microbes inhibit or enhance Th2 responses	Alters parasite survival	*T. muris* (Dea-Ayuela et al., 2008)
	Treg cells	P > M	Helminths increase Treg responses	Downregulates inflammatory responses against microbiota	
				Promotes Treg-inducing species	*H. polygyrus* (Reynolds et al., 2014)
			Helminths secrete TGF-β mimics to induce Foxp3+ Tregs	Downregulates inflammatory responses against microbiota	*H. polygyrus* and *T. circumcincta* (Grainger et al., 2010)
		M > P	Gut microbes induce Treg responses	Impacts parasite persistence and survival	*H. polygyrus* (Reynolds et al., 2014; Ohnmacht et al., 2015)
**DIRECT INTERACTIONS (NOT INVOLVING THE HOST)**					
Physical attachment	n/a	M > P	Helminth egg hatching require/is enhanced by bacteria attachment	Increases helminth colonization	*T. muris* (Hayes et al., 2010); *T. suis* (Vejzagić et al., 2015)
Heterophagy	n/a	M > P	Pathogenic bacteria phagocytosed by parasite induces virulence	Increases parasite virulence	*E. histolytica* (Galván-Moroyoqui et al., 2008)
Endosymbiosis	n/a	M > P	Enteric bacteria engulfed by parasite, but not ingested	Alters host-parasite immune interaction	*Giardia* (El-Shewy and Eid, 2004)
Secretions	n/a	P > M	Helminth body fluids/secretions have antibacterial and bacteriolytic properties	Disrupts microbiota	
		M > P	Gut microbes secrete molecules that inhibit invading parasites	Decreases parasite infections	*Cryptosporidium* (Deng et al., 2001; Foster et al., 2003; Glass et al., 2004); *Giardia* (Pérez et al., 2001); *E. tenella* (Tierney et al., 2004)
Ingestion	n/a	P > M	Helminths ingest bacteria from their gut environment	Restructures microbiota communities	*T. muris* (White et al., 2018)

*P > M: parasite effects on microbiota; M > P: microbiota effects on parasite*.

The layer of mucus that coats the gut epithelium forms a critical barrier that protects the host against pathogenic micro- and macroorganisms and mediates host interactions with all organisms in the lumen. Parasites have important effects on mucus, which may have downstream effects on the microbiota (Table 1). Many helminth infections stimulate increased mucus production, via a T helper cell type 2 (Th2) immune response in which interleukin (IL)-13 and IL-22 (Box 1) drive goblet cell proliferation and hyperplasia (Broadhurst et al., 2010). This is considered a host response that aids in worm expulsion (Hasnain et al., 2013; Turner et al., 2013). Structural changes in mucin (the glycoprotein that forms the basis of mucus) are also seen during infection with several GI nematodes (Hasnain et al., 2013; Tsubokawa et al., 2015). For example, in mice able to expel *T. muris*, there is a switch in colonic mucin expression from MUC2 to MUC5AC, which is believed to change biochemical properties of the mucus in a way that aids helminth expulsion (Hasnain et al., 2013). Some helminths also express mucin-like molecules themselves, which may play a role in host cell attachment and immune evasion (Theodoropoulos et al., 2001). The major constituent of the surface coat on *Toxocara canis* infective larvae, for example, contains a mucin-like molecule, the TES-120 protein, which may allow *T. canis* larvae to mimic the surface of endothelial cells and avoid immune recognition (Gems and Maizels, 1996). Parasitic protozoa also change intestinal mucus abundance and composition. Some species including *Entamoeba histolytica, Giardia intestinalis*, and *Tritrichomonas suis*, produce mucolytic enzymes that enable them to penetrate the mucus barrier during pathogenesis (Hicks et al., 2000; Kim and Khan, 2013). *Toxoplasma gondii* causes a general increase in the number of goblet cells, but also induces a shift in production of more acidic and neutral mucins, which is thought to increase mucus fluidity and promote parasite expulsion (Trevizan et al., 2016). Such parasite-induced changes in mucus may alter the availability of nutrients for gut microbes, microbial movement out of the gut, as well as epithelial access and attachment sites in the gut. Several microbial taxa, including species from the Bacteroidetes, Firmicutes, Actinobacteria, and Verrucomicrobia phyla (Tailford et al., 2015), use carbohydrates from mucus as a carbon source and are thus likely to gain a competitive advantage following increased mucus production. Indeed, mucin composition and glycosylation are both known to affect the abundance of mucus-utilizing taxa (Sommer et al., 2014). Several studies have now suggested that parasite-driven changes to mucus may alter the microbiota. Three studies have shown that *Trichuris* infection in rodents and pigs induces an increase in the relative abundance of the mucolytic genus *Mucispirillum* (Li et al., 2012; Holm et al., 2015; Houlden et al., 2015; Supplementary Table S1), while another study showed that the nematodes *T. muris* and *Heligmosomoides polygyrus* both increased the relative abundance of mucin-utilizing Clostridiales, whose *in vitro* growth was enhanced by the addition of mucin (Ramanan et al., 2016). A similar mechanism has been proposed to explain an increase in the relative abundance of mucolytic bacteria in *Eimeria*-infected chickens, as these protozoa also stimulate mucus production (Collier et al., 2008).

Beyond mucus, gut parasites can have important effects on the epithelial monolayer itself, with potential downstream effects on the microbiota (Table 1). For instance, helminths can increase intestinal epithelial cell (IEC) turnover (Cliffe et al., 2005), which, combined with increased mucus flow during infection, may select for gut microbes capable of replicating at a high rate to avoid being flushed from the gut. Damage to the epithelial lining is also common in parasitic infections. Many gut protozoa damage the epithelium during pathogenesis through parasite attachment, disruption of tight junctions, or cell invasion and destruction (Certad et al., 2017). In combination with disruption of the mucus layer, this damage can profoundly alter the host's interaction with their microbial flora, allowing microbes greater contact with and even translocation across the epithelial barrier, as seen during *T. gondii* (Heimesaat et al., 2006; Hand et al., 2012; Cohen and Denkers, 2014) and *Giardia* (Chen et al., 2013; Halliez, 2014) infection. In contrast, helminths often promote epithelial regeneration and mucus production through upregulation of host IL-22 production (Broadhurst et al., 2010), which helps contain bacteria within the gut and limit their access to the epithelium (Sonnenberg et al., 2012). However, some helminth species, including *Strongyloides venezuelensis* (Farid et al., 2007)*, Trichinella spiralis* (McDermott et al., 2003)*, H. polygyrus* (Shea-Donohue et al., 2001), and *Nippostrongylus brasiliensis* (Hyoh et al., 1999), have also been shown to alter junctional proteins, sometimes at sites distant from those of parasite attachment (Su et al., 2011), which can allow for the translocation of bacteria and bacterial LPS into the portal circulation (McDermott et al., 2003; Chen et al., 2005; Farid et al., 2007). Helminths may thus have opposing effects on barrier function, in that they can increase mucus production, but can also alter the epithelial monolayer in ways that facilitate microbial migration across it. The balance of these two effects as well as the rate of tissue repair they induce may determine the extent of microbial translocation that occurs with helminth infection.

**Figure 1:**
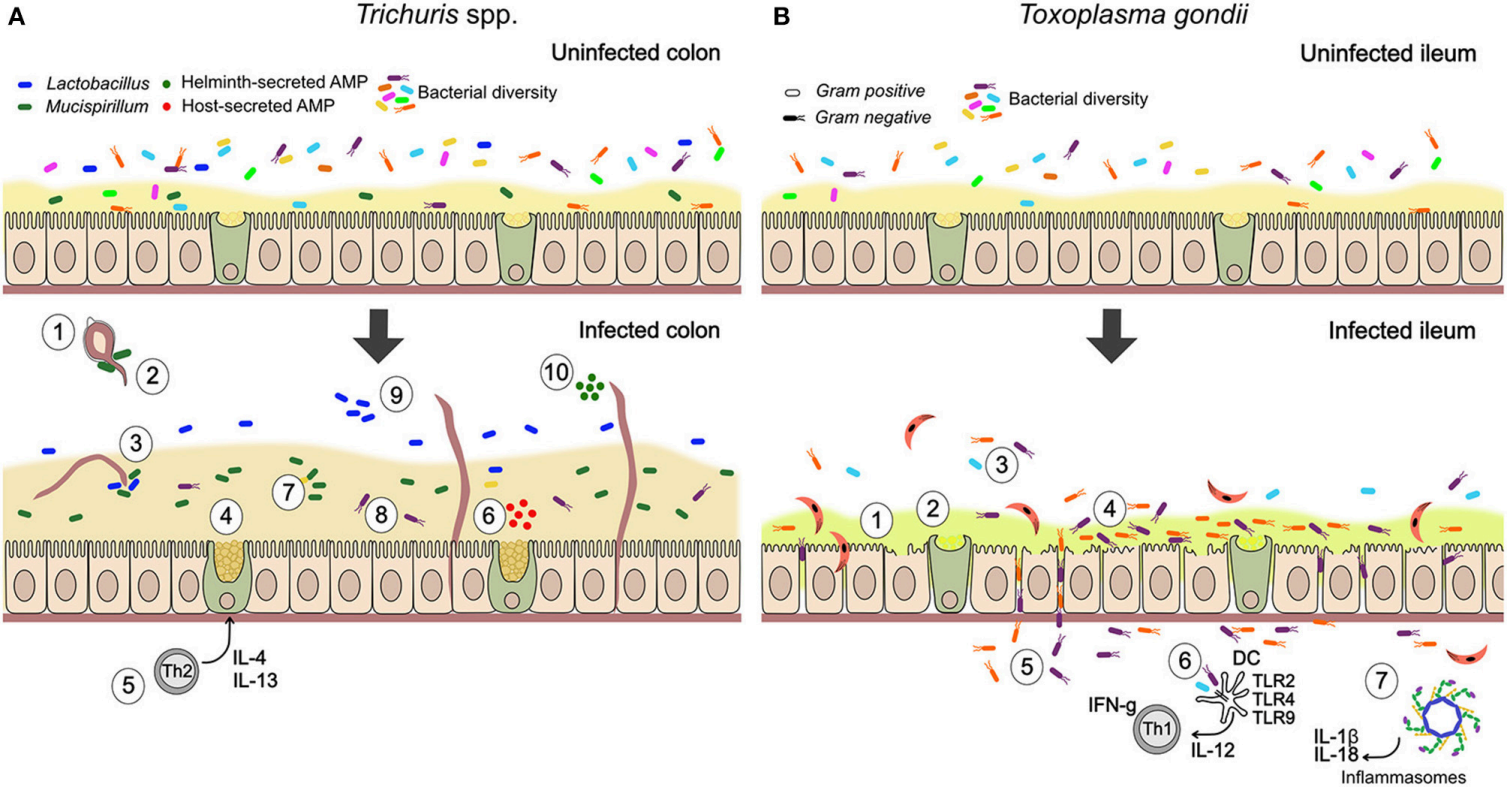
Summary of documented mechanisms by which infection with *Trichuris* spp. and *Toxoplasma gondii* may alter the gut microbiota. **(A)** In *Trichuris* infection, parasites stimulate an anti-inflammatory mucogenic immune response that may improve barrier function and reduce bacterial-driven inflammation. Direct interactions through physical attachment and secreted molecules are also likely. (1) Gut bacteria attach to polar caps and allow egg-hatching (Hayes et al., 2010). (2) *T. muris*-induced changes to the gut microbiota inhibit hatching of further *T. muris* eggs (White et al., 2018). (3) *T. muris* can ingest bacteria from their environment (White et al., 2018). (4) Mucin expression changes from MUC2 to MUC5AC, altering physical properties of the mucus (Hasnain et al., 2013). (5) *Trichuris* drives a Th2-mediated mucogenic response, leading to goblet cell hyperplasia and increase in mucus volume (Broadhurst et al., 2010, 2012). (6) Infection induces production of antimicrobial peptides (AMPs) by goblet cells (e.g., Ang4; D'Elia et al., 2009). (7) Infection drives expansion of *Mucispirillum*, a mucolytic genus of bacteria (Wu et al., 2012; Holm et al., 2015; Houlden et al., 2015). (8) Mucus can reduce adherence of bacteria to the epithelium, in the context of inflammatory disease (Broadhurst et al., 2012). (9) Infection drives expansion of *Lactobacillus* and other changes in community structure, including reduced microbial diversity (Holm et al., 2015; Houlden et al., 2015). (10) Helminths directly secrete AMPs in excretory-secretory products (Abner et al., 2001). **(B)** During *T. gondii* infection, inflammatory processes play an important role in parasite-microbiota interactions and their consequences. Degradation of barrier function by parasites allows bacterial translocation and consequent inflammation, while microbe-driven pro-inflammatory responses contribute to anti-parasite immunity. (1) Blunted villi and epithelial damage (Cohen and Denkers, 2014; Trevizan et al., 2016). (2) Change to more acidic and neutral mucins, thought to increase mucus fluidity (Trevizan et al., 2016). (3) Reduction in bacterial diversity (Heimesaat et al., 2006). (4) Increase in adherent Gram-negative bacteria (*E. coli* and *Bacteroides/Prevotella*) relative to Gram-positive (Heimesaat et al., 2006). (5) Bacterial translocation to lamina propria (Heimesaat et al., 2006; Hand et al., 2012; Cohen and Denkers, 2014). (6) Stimulation of protective pro-inflammatory immune response against parasites by bacterial TLR stimulation (Benson et al., 2009). With high dose infection, inflammatory disease (ileitis) results from a cytokine storm (Hand et al., 2012). (7) *T. gondii* infection activates NLRP1 and NLRP3 inflammasomes (Ewald et al., 2014; Gorfu et al., 2014), driving production of pro-inflammatory cytokines, IL-18 and IL-1β, that mediate resistance to the parasite but are expected to also affect gut microbes.

Together, these observations suggest that parasites can serve as ecosystem engineers for gut microbes by altering the physical landscape in which they reside. Moreover, current evidence suggests that the type of effects observed may broadly differ between parasitic helminths and protozoa. While helminths can promote barrier function and limit bacterial translocation, virulent parasitic protozoa may often have the opposite effect, degrading barrier function and allowing closer interaction between bacteria and the epithelium. This contrast is illustrated by the suite of interactions between the microbiota and two types of parasites—*Trichuris* spp. nematodes and the protozoan parasite *T. gondii*—many of which are known or thought to involve changes to epithelial barrier function (Figure 1).

To our knowledge, no studies have yet explicitly examined how microbe-driven changes to the physical gut landscape might affect parasitic infections, though such effects seem likely to occur (Table 1). The microbiota is known to affect the expression of genes involved in mucin biosynthesis (Chowdhury et al., 2007) and the strength and permeability of the colonic mucus barrier (Jakobsson et al., 2015). Since mucus is an important part of host defense against intestinal parasites by trapping them and preventing attachment to the epithelium (Hasnain et al., 2013), this is expected to have knock-on effects on parasite colonization, persistence, and fecundity. Gut microbes also alter IEC turnover (Park et al., 2016). Gram-positive commensal bacteria mediate IEC turnover and repair in the gut through the production of short-chain fatty acids (SCFAs, Box 1), with germ-free and antibiotic-treated mice showing decreased cell turnover (Park et al., 2016). Given that an increase in the rate of cell turnover can act as an “epithelial escalator” to expel nematodes such as *T. muris* (Cliffe et al., 2005), this microbial effect might be expected to reduce helminth colonization. Such interactions remain to be explored, though warrant further investigation in gnotobiotic models where microbe-driven cell turnover and mucus production can be carefully manipulated.

### Innate and adaptive immune responses

Both parasites and microbes shape the immune landscape of the gut, and can thereby select for or against particular species via top-down ecological interactions. Helminths have been shown to alter how the innate immune system responds to gut microbes by regulating the expression and responsiveness of toll-like receptors (TLRs) involved in host defense against bacterial infections (Kane et al., 2008; Semnani et al., 2008; Friberg et al., 2013; Table 1). For instance, infection of rats with *Hymenolepis diminuta* increased expression of TLR2 and TLR4 in the jejunum and colon (Kosik-Bogacka et al., 2012), and *H. polygyrus* infection in mice induced TLR4 expression in lamina propria T cells (Ince et al., 2006). While the upregulation of TLRs typically induces pro-inflammatory cytokines against bacterial infection, interestingly, TLR4-induced mucosal T cells in *H. polygyrus* infected mice instead led to the production of transforming growth factor-beta (TGF-β) (Box 1), a cytokine important in promoting T cell tolerance (Ince et al., 2006). This may serve as a mechanism by which helminths regulate mucosal inflammation against gut microbes and thus maintain immune homeostasis in the gut. Some gut parasites also affect host production of antimicrobial peptides (AMPs) (Table 1). *Nippostrongyloides brasiliensis* infection upregulates angiogenin 4 (Ang4) and downregulates regenerating islet-derived protein 3 gamma (RegIIIγ) and lysozymes (Lyz1 and Lyz2), all of which have bactericidal activity against a range of Gram-positive and Gram-negative bacteria (Fricke et al., 2015). *T. muris* similarly increases Ang4 production in colonic goblet cells (D'Elia et al., 2009; Figure 1), and *H. polygyrus* infection increases expression levels of RegIIIγ in the murine cecum (Su et al., 2014). Since host TLR responses and AMPs have clear microbicidal effects, such helminth-driven changes are expected to impact the gut microbiota composition. However, importantly, we still do not understand how and why such parasite-microbe cross-immunity occurs. One possibility is that changes to TLR expression and AMP secretion are simply a downstream consequence of parasite-induced changes to the microbiota. Alternatively, such changes may reflect a parasite adaptation that enhances its own fitness. For example, upregulation of specific TLRs (e.g., TLR2 and TLR5) has been shown to expand regulatory T (Treg) cells (Crellin et al., 2005; Liu et al., 2006; Sutmuller et al., 2006), which may aid helminth survival within hosts. Such parasite-driven TLR and AMP responses could also represent a host adaptation, if these effectors are anti-parasitic as well as anti-microbial, or if hosts use parasitic infection as a cue to protect themselves against subsequent microbiota-driven pathologies. For example, if helminth infection can allow translocation of bacterial LPS into the portal circulation resulting in systemic endotoxemia (Farid et al., 2007), a host response to helminths that includes AMP production could protect the host against harmful immune responses elicited by microbial contact, and thus maintain immune homeostasis. Ang4 induction by *T. muris* is an interesting case, as this phenotype is associated with host resistance (D'Elia et al., 2009). This begs the question whether Ang4 aids worm clearance by directly acting on *T. muris* (as Ang4 has been linked to toxicity toward GI parasites, Hamann et al., 1987), or by specific helminth-microbiota interactions occurring only in resistant mouse strains. Experiments in germ-free and gnotobiotic mice could shed valuable light on these hypotheses and the evolutionary underpinnings of such effects.

Inflammasome activation represents another potentially important innate immune mechanism mediating gut parasite-microbiota interactions that warrants further study. Inflammasomes are protein complexes that sense pathogen and endogenous danger signals in the cytosol and induce inflammation through the secretion of pro-inflammatory cytokines such as IL-18 and IL-1β (Martinon et al., 2002). Inflammasome deficiency has been associated with gut microbial dysbiosis and the induction of an exaggerated autoinflammatory response due to a disrupted barrier function (Elinav et al., 2011). Various parasites affect inflammasome activation, which may in turn alter the microbiota. For example, *Fasciola hepatica* secretes a peptide (FhHDM-1) that inhibits NLRP3 inflammasomes (Alvarado et al., 2017), whereas both schistosomes and *H. polygyrus* excretory-secretory (ES) products lead to the activation of NLRP3 inflammasomes (Ritter et al., 2010; Zaiss et al., 2013; Meng et al., 2016). *T. gondii* infection activates NLRP1 and NLPR3 inflammasomes, which limits parasite load and dissemination (Ewald et al., 2014; Gorfu et al., 2014), while a murine commensal protozoan, *Tritrichomonas musculis*, was also shown to activate inflammasomes, leading to protection against *Salmonella typhimurium* infection (Chudnovskiy et al., 2016). Inflammasome-induced epithelial IL-18 secretion may be a particularly important factor shaping gut microbial communities, as it has been shown to prevent colonization of the murine gut by colitogenic microbes (Elinav et al., 2011). Conversely, microbes may also affect parasites via inflammasome activation. Microbiota-derived metabolites have been shown to activate NLRP6 inflammasomes (Levy et al., 2015), which may lead to a pro-inflammatory environment conducive to clearance of protozoan parasites (Ewald et al., 2014; Gorfu et al., 2014), but also potentially increased chronicity of helminth infections (Zaiss et al., 2013).

Interactions between parasites and the microbiota also likely involve adaptive immune responses. Many gut nematodes and some members of the microbiota promote a regulatory immune environment through the induction of Treg cells, raising the intriguing possibility that these groups may interact mutualistically and promote each other's persistence and growth in the gut. *H. polygyrus* and *Teladorsagia circumcincta* secrete TGF-β mimics that exploit the host's own regulatory mechanisms to induce Foxp3+ Treg cells (Grainger et al., 2010), and various intestinal bacteria including *Bifidobacterium infantis* (O'Mahony et al., 2008), *Bacteroides fragilis* (Round and Mazmanian, 2010), *Clostridium* spp. (Atarashi et al., 2011, 2013; Narushima et al., 2014), and *Lactobacillus* spp. (Smits et al., 2005; Jang et al., 2012) also induce tolerogenic Treg responses. Recent empirical studies provide support for the idea that these parasitic and bacteria species may promote each other's persistence in the gut. Ohnmacht et al. (2015) demonstrated that mice lacking microbiota-inducible RORγt+ Tregs had stronger Th2 immunity and hence greater resistance to *H. polygyrus* infection, implying that Treg induction by the microbiota ordinarily promotes *H. polygyrus* persistence (Ohnmacht et al., 2015). Reynolds et al. (2014) further provided evidence for positive mutual reinforcement between a Treg-promoting helminth and a species of *Lactobacillus*. *H. polygyrus* increased *Lactobacillus taiwanensis* abundance in the murine gut, and administration of *L. taiwanensis* increased *H. polygyrus* burdens (Reynolds et al., 2014). Further studies have also suggested *H. polygyrus* promotes Treg-inducing species of *Lactobacillus* (Rausch et al., 2013; Kreisinger et al., 2015; Supplementary Table S1). Thus, it seems gut-dwelling helminths and some bacteria may synergise to create a tolerogenic environment, in which disruption to either community may affect tolerance to the other and hence, consequent disease. Co-infection experiments have also shown that helminth-induced T cell responses can inhibit the growth of bacterial pathogens (Graham, 2008; Salgame et al., 2013). For example, Chen et al. (2005) showed that *H. polygyrus* infected mice were more susceptible to *Citrobacter rodentium* infection and pathology. This effect was abrogated in STAT6-deficient mice and hence dependent on Th2-type immunity, and thought to reflect Th2-driven impairment of protective T helper cell type 1 (Th1) immune responses (Chen et al., 2005). Microbial enhancement of Th1 immunity may also allow increased growth and survival of gut helminths. Suggestive evidence for this comes from a recent study in which laboratory C57BL/6 mice were moved to a more natural, farm-like environment (Leung et al., 2018). This environmental transition increased gut microbial diversity and altered microbiota composition, while skewing immunity toward a Th1 response and rendering mice more susceptible to subsequent *T. muris* infection. The authors hypothesized that environmental driven changes to the gut microbiota likely contributed to the Th1 immune bias, which in turn increased susceptibility to *T. muris* infection.

While there is a growing literature on immune-mediated interactions between helminths and the microbiota, fewer studies have investigated immune-mediated interactions between protozoan parasites and the microbiota, with the vast majority considering how microbes may alter anti-parasite immunity, but not the reverse. Several studies have shown that members of the gut microbiota can enhance immune responses to parasitic protozoa. Benson et al. (2009) showed that both mucosal innate and adaptive immune responses against orally administered *T. gondii* relied on indirect stimulation of dendritic cells by the gut microbiota through TLR2, TLR4, and TLR9 signaling (Benson et al., 2009; Figure 1). More recently, Burgess et al. (2014) showed that colonization of mice by segmented filamentous bacteria (SFB) reduces susceptibility to *E. histolytica* infection (Burgess et al., 2014). Intriguingly, this protection was largely mediated by extra-intestinal effects of SFB on immune cell development, as transfer of bone marrow-derived dendritic cells (BMDCs) from SFB-colonized mice was capable of conferring protection. BMDCs from SFB-colonized mice produced more IL-23 in response to *E. histolytica* parasites (as well as in response to LPS, suggesting the priming effect is not specific to *E. histolytica*), apparently driving stronger intestinal IL-17A (Box 1) induction and increased neutrophil abundance, important components of a protective immune response against this parasite. Exactly how signaling from the microbiota can alter immune cell development in the bone marrow remains unclear, though there is some evidence suggesting that bacterial soluble mediators may alter granulopoiesis though epigenetic mechanisms (Burgess et al., 2016).

### Direct interactions

While host interactions with both gut parasites and microbes clearly provide ample opportunity for these groups of organisms to indirectly influence one another, several examples illustrate how prolonged coexistence in the gut has also driven the evolution of direct interaction mechanisms (not involving the host), both positive and negative. For instance, *in vitro* studies have demonstrated that some helminths rely on microbial cues to initiate the vertebrate stage of their life cycle. Eggs of the colon-dwelling nematode *T. muris* can only hatch when gut microbes directly attach to the polar egg caps, stimulating the release of infective larvae (Hayes et al., 2010; Figure 1, Table 1). Other forms of direct microbial interaction may also underlie the increased resistance of germ-free (Box 1) animals to colonization with other helminths, including the rodent nematodes *H. polygyrus* (Wescott, 1968; Chang and Wescott, 1972), *T. spiralis* (Przyjalkowski, 1968; Przyjalkowski and Wescott, 1969), and *N. brasiliensis* (Wescott and Todd, 1964), and *Ascaridia galli* in chickens (Johnson and Reid, 1973), though this possibility has not been explicitly explored. Seemingly adaptive direct interactions with the microbiota are also evident among protozoa. For example, phagocytosis of enteropathogenic bacteria (e.g., *Escherichia coli* and *Shigella dysenteriae*) by *E. histolytica* induces a switch to virulence in the parasite, allowing it to invade the host epithelium and cause dysentery (Galván-Moroyoqui et al., 2008; Table 1).

Other effects of gut microbes on parasites suggest a more adversarial evolutionary history. Cell-free supernatants of certain probiotic bacteria have been shown to have inhibitory effects on the protozoan parasites *Crytosporidium* (Deng et al., 2001; Foster et al., 2003; Glass et al., 2004), *Giardia* (Pérez et al., 2001), and the chicken parasite *Eimeria tenella* (Tierney et al., 2004), suggesting that some gut bacteria secrete molecules that directly attack sympatric eukaroyotes (Table 1). Similarly, some helminths produce anti-bacterial secretions (Table 1). Body fluids of the pig roundworm, *Ascaris suum*, have antibacterial and bacteriolytic properties (Wardlaw et al., 1994; Kato, 1995). *Trichuris suis* ES products have antibacterial activity *in vitro* (Abner et al., 2001; Figure 1), and *H. polygyrus* ES products contain at least 8 lysozyme homologs (Hewitson et al., 2011) that can potentially degrade peptidoglycan in bacterial cell walls. Several helminths also secrete peptides termed helminth defense molecules (HDMs), which are similar to human AMPs and have not only direct bactericidal effects, but also immunomodulatory properties (Cotton et al., 2012). Perhaps the best characterized HDM is FhHDM-1 secreted by the trematode *F. hepatica*, which binds LPS, preventing it from stimulating a strong inflammatory response (Robinson et al., 2011). Thus, these helminths appear to secrete peptides that not only directly kill microbes, but also alter host immune responses toward them, presumably to enhance their own colonization and persistence. Future studies are needed to characterize helminth secretions and their effects on the natural gut microbiota, to explore whether gut-dwelling protozoa also secrete anti-bacterial molecules, and to understand how and why these apparent weapons against cohabiting gut bacteria evolved.

It is also quite possible that parasites and gut microbes interact through overlapping resource requirements, either competing for the same nutrients or cross-feeding (Box 1), whereby members of one species utilize waste products of another. Finally, eukaryotic parasites may also simply prey upon prokaryotes (e.g., a helminth grazing upon, or an amoeba phagocytosing, gut bacteria). A study by White et al. (2018) recently demonstrated that *T. muris* gains its microbiota from the murine intestine it infects, likely via ingestion of bacteria from the gut environment (White et al., 2018; Figure 1; Table 1). In turn, these changes to the murine microbiota further inhibited egg hatching during secondary *T. muris* infection (White et al., 2018; Figure 1). These types of mechanisms have received little attention to date compared with immune-mediated effects, perhaps because parasite and microbial nutrient and microhabitat requirements are often poorly understood (Sukhdeo and Bansemir, 1996). However, resource-based interactions between intestinal parasites and microbes is an area of study ripe for future investigation. This could involve for example metabolomic or proteomic studies to explore whether parasite ES components contain substrates for microbial metabolism and vice versa, and *in vitro* co-culture approaches where feasible.

## Consequences of parasite-microbiota interaction for host health and disease

Perturbations to the microbial and parasitic communities of the gut contribute to a variety of health problems and diseases. Emerging insights into key mechanisms underpinning parasite-microbiota interactions, as discussed above, should lead to a better understanding of variable parasite infection outcomes, and, ultimately, to predicting the consequences of gut community perturbations for host health. This is beginning to be borne out by studies demonstrating the health implications of parasite-microbiota interactions via a range of pathways, as discussed below.

### Inflammation and commensal tolerance

Many parasites, both helminth and protozoan, have important effects on gut inflammation. Helminths are often credited with ameliorating inflammatory disease including the various forms of inflammatory bowel disease (Summers et al., 2005a,b). Several studies have now provided evidence that protective effects of helminths against inflammatory disorders may be partially mediated by their effects on the microbiota. A study in rhesus macaques with idiopathic chronic diarrhea (ICD) found that infection with *Trichuris trichiura* ameliorated symptoms of ICD and resulted in concomitant reductions in bacterial attachment to the colonic epithelium and altered epithelia-associated gut microbial communities (Broadhurst et al., 2012; Figure 1). This was thought to be due to Th2-driven activation of mucus production, which improved barrier function and reduced the ability of bacterial pathobionts to attach and drive inflammation (Broadhurst et al., 2012; Wolff et al., 2012; Table 1). Elucidating the exact role of the microbiota in these kinds of effects is experimentally challenging, but studies in laboratory animals have begun to successfully tackle this. A recent study by Ramanan et al. (2016) provides perhaps the first attempt to isolate the impact of parasite-microbiota interactions on inflammatory disease. This study showed that two species of intestinal nematodes, *T. muris* and *H. polygyrus*, reduced ileitis in *Nod2*^−/−^ mice by inhibiting the colonization of an inflammatory commensal species, *Bacteroides vulgatus* (Ramanan et al., 2016). Intriguingly, the protective effect was transferable through co-housing, which allowed for microbial, but not parasite transmission: uninfected *Nod2*^−/−^ mice that were co-housed with helminth-infected counterparts had reduced *B. vulgatus* levels and ameliorated inflammatory disease, providing evidence for a causal role of the gut microbiota in these effects. Their findings again suggested a key role for the helminth-induced Th2 mucogenic response, but also demonstrated that microbe-microbe interactions were at play, since *T. muris* infection increased the growth of Clostridia strains that were capable of inhibiting *B. vulgatus* colonization of the epithelium. Experiments with mono- or poly-colonized gnotobiotic mice could shed further light on the precise microbes involved in such effects.

In contrast to helminths, several protozoan parasites are known to either induce or exacerbate intestinal inflammatory disease (Wilhelm and Yarovinksy, 2014; Buret et al., 2015), with interactions with the gut flora again being implicated. For others, such as *Blastocystis hominis*, the picture is less clear, and effects on inflammatory disease may be context-dependent, including potential dependence on interactions with the gut microbiota (Partida-Rodríguez et al., 2017). Many parasitic protozoa live in the lumen for long periods without causing detectable harm. Pathogenesis typically involves attachment to and sometimes invasion of the epithelium, triggering inflammatory immune responses. These virulence-associated processes can allow translocation of ordinarily tolerated commensals across the epithelial barrier, further driving inflammatory disease. For example, parasite-induced changes to host-commensal interactions have been implicated in post-infectious irritable bowel syndrome (IBS) caused by *Giardia lamblia*. Recent work has revealed that *Giardia* infection causes persistent tight junctional damage, bacterial penetration, and mucosal inflammation that continues even after parasite clearance, potentially driving post-infectious IBS (Chen et al., 2013; Halliez, 2014). Microbiota involvement in *T. gondii*-induced ileitis has also been known for some time. High dose infection leads to ileal inflammation in mice, with mucosal invasion of the lamina propria by adherent and invasive pathobionts (e.g., *E. coli* and *Bacteroides*/*Prevotella*) accompanied by gut microbial compositional changes, including a reduction in diversity and a switch from Gram-positive to Gram-negative bacteria (Heimesaat et al., 2006; Craven et al., 2012; Cohen and Denkers, 2014; Figure 1). Prior administration of antibiotics protects mice from this parasite-induced inflammation, and gnotobiotic mice are resistant to it (Heimesaat et al., 2006), demonstrating the microbiota's role in this effect. Exactly how *T. gondii* drives loss of tolerance to gut microbes and dysbiosis remains unclear, but studies suggest that parasite-microbiota interactions involving Paneth cells may be important (Raetz et al., 2013; Cohen and Denkers, 2014). Interestingly, similar microbial translocation due to human hookworm infections has been documented, but was not associated with systemic inflammatory responses (George et al., 2012). This may be attributed to counter-regulatory measures induced by hookworms, such as increased IL-10 (Box 1) production, that decrease mucosal inflammation. Thus, the emerging view is that gut helminths and invasive protozoa often have opposite effects on intestinal inflammatory disease, and that infection-induced changes to barrier function and bacterial translocation play an important role in these effects.

### Colonization resistance

Colonization resistance (CR) (Box 1) refers to the phenomenon by which the microbiota protects against pathogens (usually bacterial and viral pathogens are considered under CR). While classic co-infection studies have shown that gut-dwelling parasites can alter susceptibility to gut bacterial infection, until recently most work has assumed this occurs via mechanisms not involving the commensal microbiota. For example, by driving an anti-inflammatory gut environment, helminths can antagonize bacterial pathogens that thrive under inflammation, such as *Salmonella enterica* Serovar Typhimurium (Lupp et al., 2007; Stecher et al., 2007; Winter et al., 2010), or conversely promote pathogens that might otherwise be effectively controlled by inflammation (Chen et al., 2005; Su et al., 2014). However, it is also possible that parasitic effects on gut bacterial infections are at least partially mediated by their impact on the microbiota and microbe-microbe interactions. A number of intestinal helminth species have been shown to promote the expansion of species belonging to the genus *Lactobacillus* (Walk et al., 2010; Osborne et al., 2014; Reynolds et al., 2014; Fricke et al., 2015; Holm et al., 2015; Supplementary Table S1), which are often considered probiotic and have been shown to promote CR (Bäumler and Sperandio, 2016). For example, *Lactobacillus delbrueckii* reduces the cytotoxicity of *Clostridium difficile* and the adherence of *C. difficile* to colonic cells *in vitro* (Banerjee et al., 2009). Thus, helminth infection might be expected to enhance CR by promoting the expansion of *Lactobacillus*.

Several studies have also shown that parasite infection can alter gut microbial diversity. Since low microbiota diversity is thought to be an important factor in the breakdown of CR and the etiology of inflammatory disorders (Ott et al., 2004; Sepehri et al., 2007), such effects could have knock-on effects on pathogen susceptibility. The direction in which helminths alter gut microbial diversity varies across species and contexts, however (Supplementary Table S1). *T. muris* infection reduces microbiota diversity in wild-type mice (Holm et al., 2015; Houlden et al., 2015), but increases diversity in *Nod2*^−/−^ mice with ileitis (Ramanan et al., 2016). Interestingly, several studies in humans have also reported increased gut microbial diversity in individuals infected by gut helminths (Lee et al., 2014; Ramanan et al., 2016) or protozoa (Morton E. R. et al., 2015; Audebert et al., 2016), or changes in diversity following parasite treatment (Yang et al., 2017). While it is too early to assert any general patterns as to how particular parasite species alter microbiota diversity, if diversity *per se* alters CR (Lozupone et al., 2012), these early findings suggest parasites could affect CR through impacts on microbiota diversity. Similarly, if microbiota composition affects CR (e.g., as suggested for *C. difficile*), the growing number of studies showing that parasites alter microbiota composition (Supplementary Table S1) suggests this may have knock-on effects on CR.

### Immune homeostasis beyond the gut

Parasite-microbiota interactions may have wider impacts on immune homeostasis beyond the gut. For example, parasite-induced disruptions of the gut epithelial barrier could drive systemic breakdown of immune homeostasis in the form of sepsis and even septic shock. Less dramatically, parasite-microbiota interactions may alter host susceptibility to non-gastrointestinal allergies and autoimmune disorders. Several nematodes promote gut bacteria that produce SCFAs, which travel throughout the body and regulate systemic immune responses (Honda and Littman, 2016). Zaiss et al. (2015) recently showed that the nematode *H. polygyrus* protects against airway inflammation in a mouse model of asthma, and that this was at least partially modulated by changes to the microbiota and SCFA production. This amelioration of asthma due to changes in the gut microbiota and SCFA levels was similar to that seen when mice are fed a high fiber diet (Trompette et al., 2014). *H. polygyrus* infection led to an expansion of Clostridiales (also documented by Ramanan et al., 2016) and in cecal SCFA concentrations. Microbiota transfer from helminth-infected mice also conferred protection against allergic airway inflammation, in a manner that was dependent on the SCFA G-protein receptor GPR41 (Zaiss et al., 2015). Several other nematode species, including *A. suum* in pigs and *Necator americanus* in humans, also tended to increase SCFA levels in the gut (Zaiss et al., 2015), suggesting SCFA increases may be a common feature of helminth infection. Thus, although it is undoubtedly true that nematodes have some direct immunomodulatory effects through secreted proteins (McSorley et al., 2013), at least part of their immunomodulation seems to be microbially mediated. There is now great interest in understanding the extent to which well-known immunomodulatory effects of helminths may be mediated by effects on the microbiota (Giacomin et al., 2015; Gause and Maizels, 2016). While various incarnations of the hygiene hypothesis have implicated either disruptions to the commensal flora or reduced helminth exposure in the rise of autoimmunity and allergy, interactions between the two may be an important piece of this puzzle (Loke and Lim, 2015). The emerging view is that immune homeostasis is a complex trait with inputs from several elements of the gut ecosystem including gut microbes, eukaryotic parasites, and diet, which all interact with one other to shape host health and disease.

### Host metabolism and nutrition

Host metabolism is regulated to a large extent by the gut microbiota, as many complex carbohydrates cannot be degraded by host enzymes alone (Flint et al., 2012). Parasite-induced changes to the microbiota may thus alter the extent to which this community provides nutrition to the host. Several studies of *Trichuris* nematodes have documented reduced carbohydrate metabolism upon infection (Li et al., 2012; Wu et al., 2012; Lee et al., 2014) or a reduction in the breakdown products of plant-derived carbohydrates (Houlden et al., 2015; Figure 1), which coincided with declines in the relative abundance of cellulolytic or other carbohydrate utilizing bacteria, including *Ruminoccocus, Prevotella, Roseburia*, and *Parabacteroides* (Supplementary Table S1). These findings suggest that helminth-induced malnutrition may be at least partly mediated via negative effects on fermentative microbes, potentially contributing to the weight loss caused by this parasite (Houlden et al., 2015). On the other hand, *H. polygyrus* infection has been linked with increased microbial carbohydrate metabolism, both in laboratory (Zaiss et al., 2015) and wild (Kreisinger et al., 2015) rodents. Infection by *Haemonchus contortus* in ruminants has also been suggested to increase gut microbial protein metabolism, allowing the host to partially compensate for infection-driven impaired protein metabolism (Li et al., 2016). Anatomical differences affecting digestion between monogastric and ruminant organisms, however, should be considered when comparing how parasite-microbiota interactions impact metabolism. Thus, parasites may alter the host's ability to extract and utilize energy from their food via microbial metabolism, a possibility that requires further investigation. Existing helminth studies indicate the form of such effects is likely to vary among species.

Gut parasite-microbiota interactions may also play a role in the complex etiology of human malnutrition. Gut helminths (Ezenwa, 2004), protozoa (Mondal et al., 2012), and more recently the microbiota (Gordon et al., 2012; Kane et al., 2015) have all been implicated as part of the complex web of factors underpinning undernutrition. Two recent studies on severe and moderate acute malnutrition in Malawian and Bangladeshi children have shown that these conditions are characterized by a relatively “immature” microbiota (Smith et al., 2013; Subramanian et al., 2014), with transfer experiments in gnotobiotic mice showing that community composition plays a causal role in malnutrition (Smith et al., 2013). At the same time, diarrhea-causing gut protozoan infections have also been implicated in the etiology of malnutrition. Repeated diarrhea is an important risk factor for stunting (Checkley et al., 2008; Richard et al., 2013), and environmental enteric dysfunction (EED), characterized by villous atrophy and chronic inflammation, is a hallmark of undernutrition, which is thought to be driven by repeated enteric infection (Prendergast and Humphrey, 2014). Mouse studies have shown that parasites like *Giardia* and *Cryptosporidium* are capable of causing a similar syndrome in mice, with growth impairment and enteropathy (Coutinho et al., 2008; Bartelt et al., 2013). The question then arises, to what extent might the effect of diarrheal disease on malnutrition arise via parasite-induced shifts in the microbiota? Interestingly, Subramanian et al. (2014) recently showed that diarrheal episodes in children from the Mirpur slum of Dhaka, Bangladesh (known from previous work to be commonly caused by gut protozoal infections Mondal et al., 2012), were accompanied by shifts in the microbiota toward a more “immature” phenotype (Subramanian et al., 2014), and Smith et al. (2013) showed that such immature microbial communities can drive acute malnutrition in mice (Smith et al., 2013). Future studies investigating the role of parasite-driven gut microbial dysbiosis in malnutrition are therefore warranted.

## Applications of understanding parasite-microbiota interactions

Understanding the mechanisms and consequences of gut parasite-microbiota interactions might lead to improved treatment for parasitic infections and dysbiosis of the microbiota. For instance, administration of carefully designed probiotics (Box 1) or prebiotics (Box 1) could improve treatment of intestinal parasite infections (Rastall et al., 2005). Probiotics, including species of *Lactobacillus* and *Bifidobacterium* (Butel, 2014), are thought to protect against infection by competing with pathogens for space and resources, modulating the host immune system, and producing antimicrobial peptides (Corr et al., 2009). Although studies investigating the effects of probiotics on helminth infections have yielded conflicting results, with studies showing that probiotics can reduce helminth burdens (Stefanski and Przyjalkowski, 1965, 1966; Bautista-Garfias et al., 1999, 2001; Santos et al., 2004; Basualdo et al., 2007; Martínez-Gómez et al., 2009, 2011; Chiodo et al., 2010; Oliveira-Sequeira et al., 2014), increase susceptibility to helminths (Dea-Ayuela et al., 2008; Reynolds et al., 2014), or have no effect on helminth burden (de Waard et al., 2001; Verdú et al., 2004), the majority of published studies have found a decrease in helminth burdens with probiotic administration. This suggests that certain probiotic species may help protect against helminth infection, though the underlying mechanisms remain unclear. Several studies suggest protection by *Lactobacillus* may involve the induction of an effective Th2 response (Martínez-Gómez et al., 2011) or helminth specific antibodies (Martínez-Gómez et al., 2009). Similarly, several *Lactobacillus* strains have been shown to inhibit protozoan infection, for example by stimulating a humoral immune response against *Giardia* (Benyacoub et al., 2005) and reducing adherence of *Giardia* trophozoites to the mucosal surface (Shukla et al., 2008). Further studies are therefore needed to explore how specific probiotic species improve host immune responses against parasitic infections. Elucidating the mechanistic basis of such effects should also improve predictability across microbial species, strains, and parasites.

As an alternative to probiotics, with sufficient understanding of both parasite-microbiota interactions and dietary effects on microbes, further use could be made of dietary prebiotics as anti-parasite treatments. Prebiotics could be designed to stimulate growth of specific gut microbes that inhibit parasites or reduce their virulence, such that simple dietary changes could constitute an effective intervention against parasitic infection. Several studies suggest such an approach can work. The addition of inulin, a complex polysaccharide metabolized by a restricted group of microbes, to the diet of pigs has been shown to dramatically reduce infection by the helminths *Oesophagostomum dentatum* and *T. suis* (Petkevicius et al., 1999, 2001, 2003, 2007; Thomsen et al., 2005). To what extent such effects are mediated by changes in gut microbes or are simply a result of changes to the physical gut landscape that may impact helminth success (e.g., expulsion rate out of the gut) remains to be elucidated. Nonetheless, such findings raise the possibility that prebiotics could be an effective means of reducing parasite susceptibility via manipulation of the gut microbial community.

Increased understanding of parasite-microbiota interactions will also be essential for predicting the wider health consequences of specific anti-parasite and antibiotic treatments. Experimental studies suggest anthelmintic treatments may alter host susceptibility to other bacterial and protozoan infections (Knowles et al., 2013; Pedersen and Antonovics, 2013; Ezenwa and Jolles, 2015), potentially by removing helminth immunomodulatory effects or competitive interactions with other species. Similarly, perturbing parasite communities via drug treatments may have knock-on effects on the gut microbiota, but such studies are just beginning to emerge. For example, in mice infected with *T. muris*, anthelmintic treatment reversed the declines in microbiota diversity and compositional changes observed with experimental infection (Houlden et al., 2015), and in helminth-infected humans given albendazole, overall microbiota diversity decreased, with a reduction in Clostridiales and expansion of Bacteroidales (Ramanan et al., 2016). However, for most anti-parasite treatments, nothing is known about impacts on the microbiota, let alone how the timing, frequency, and dose of treatment may influence this, and how long-lasting or reversible such perturbations may be. Similarly, antibiotic treatment may also alter the susceptibility of hosts to parasitic infections through parasite-microbe interactions. For instance, antibiotic administration in immunodeficient mice decreased aerobic and anaerobic microbes in the gut and resulted in significantly reduced numbers of hatched *T. muris* eggs and resulting worm burdens (Hayes et al., 2010). Treating mice with a low-dose antibiotic also increased susceptibility to *H. polygyrus* infection without causing significant reductions in bacterial abundance (Reynolds et al., 2014). On the one hand, these sorts of indirect effects call for treatment studies to adopt a community ecology framework and explicitly examine effects of treatment on the wider GI community, enabling us to protect against negative unintended consequences of treatment at the ecosystem level. Simultaneously, they also raise interesting possibilities for the use of drugs targeting disease caused by one organism to be used in tackling another, even from another domain of life.

## Future directions

### Experimental tools and approaches

Most current research investigating effects of parasites on the microbiome takes advantage of next generation sequencing technology, with a typical study testing for broad changes in gut bacterial community composition upon parasite infection using 16S ribosomal RNA (rRNA) amplicon sequencing. 16S rRNA sequencing is a PCR-based method in which primers targeting highly variable gene regions are used to amplify bacterial DNA in a sample, and the resultant pool of sequences analyzed to assess taxon relative abundance. While this method provides an important first step toward understanding parasite-microbiota interactions, we now need to look beyond the broad taxonomic changes it can reveal to probe mechanisms further and better predict infection outcomes. For example, an increase in the genus *Bacteroides* following infection could have various effects on hosts depending on the context and particular species involved. A move toward understanding the functional and metabolic roles of gut microbes altered by parasitic infection, in addition to their interactions with other microbial species, is now needed. A range of methods besides 16S rRNA sequencing are available for characterizing the microbiome, that could be useful in this context (reviewed in Fraher et al., 2012). In Table 2, we summarize the various approaches available for probing parasite-microbiota interactions, together with their uses and limitations. Experiments with mono- or poly-colonized gnotobiotic animals are likely to prove particularly fruitful for probing mechanisms, as they can test how particular microbes modulate infection traits and health outcomes. When paired with manipulation of host traits such as specific immune pathways using knockout or transgenic animals, such experimental approaches provide a powerful toolkit for investigating the mechanisms and health consequences of parasite-microbiota interactions. Tools from community ecology are also likely to be useful for understanding species interactions in complex biological communities. For example, community perturbation experiments (Box 1) involving anti-parasite or antibiotic treatments can complement controlled infection experiments, providing a powerful way to examine how alteration of one group of organisms affects another over time.

**Table 2:** Uses and limitations of different approaches for studying parasite-microbiota interactions.

**Approach**	**What this approach tell us and its advantages**	**Limitations**	**Examples of experimental approach with helminths**
Controlled parasite infection in conventional animals	•Can inform about how a parasite alters a diverse gut microbiota, while allowing control of key factors such as host and parasite genotype, infection dose, diet, and environment •When combined with manipulation of host genetics (e.g., knock-outs or transgenic animals), can inform on mechanistic basis of effects	•Does not accurately reflect conditions in natural populations •Often difficult to precisely dissect mechanisms, without simultaneous manipulation of host genetics	Walk et al., 2010; Broadhurst et al., 2012; Li et al., 2012; Wu et al., 2012; Plieskatt et al., 2013; Rausch et al., 2013; Reynolds et al., 2014; Fricke et al., 2015; Holm et al., 2015; McKenney et al., 2015; Zaiss et al., 2015; Wegener Parfrey et al., 2017; Leung et al., 2018
Controlled parasite infection in germ-free animals	•Can inform about how the presence of any gut bacteria affects a phenotype of interest (e.g., parasite colonization, reproduction, or survival, or parasite-mediated effects on host health) •When combined with manipulation of host genetics (e.g., knock-outs or transgenic animals) can inform on mechanistic basis of such effects	•Germ-free animals experience extensive immune defects, such that interpretation of findings with respect to immune-mediated interactions can be challenging •Costly to maintain germ-free facilities and perform experiments	Wescott and Todd, 1964; Wescott, 1968; Weinstein et al., 1969; Chang and Wescott, 1972; Johnson and Reid, 1973
Controlled parasite infection in gnotobiotic animals	•Can inform about how specific single gut microbes, simple defined microbial communities, or particular complex microbial communities of interest affect a phenotype of interest (e.g., parasite colonization, reproduction, or survival, or parasite-mediated effects on host health)	•Mono-colonized or gnotobiotic mice with very simple communities may retain some of the immune defects of germ-free animals •Costly to maintain germ-free facilities and perform experiments	Przyjalkowski, 1968; Przyjalkowski and Wescott, 1969; Johnson and Reid, 1973
Administration of probiotics	•Can inform about whether particular bacterial species or strains can protect against (or exacerbate) parasite infection	•Probiotics do not always colonize or stably persist in the gut •Commercially available probiotics may not be the most relevant to the system in question, in which case, custom probiotics (and bacteria-free placebo) need manufacturing	Stefanski and Przyjalkowski, 1965, 1966; Bautista-Garfias et al., 1999, 2001; de Waard et al., 2001; Santos et al., 2004; Verdú et al., 2004; Basualdo et al., 2007; Dea-Ayuela et al., 2008; Martínez-Gómez et al., 2009, 2011; Chiodo et al., 2010; Oliveira-Sequeira et al., 2014; Reynolds et al., 2014
*In vitro* studies	•Can inform about whether particular microbes or their secretions affect parasites, or whether a particular parasite or their secretions affect bacteria, in the absence of a host •Can examine whether interactions observed *in vivo* have a host-independent (direct) component	•Requires a suitable *in vitro* system to examine microbial growth and/or parasite traits (e.g., survival, invasion, or virulence), which can be hard to establish •*In vivo* relevance of *in vitro* assays or findings may not be clear	Hayes et al., 2010; Vejzagić et al., 2015
Observational studies in natural populations	•Can detect associations between parasites and microbial community composition or diversity in a natural setting that could reflect within-host interactions	•Hard to detect causal interactions from correlational data. Confounding factors may drive Type 1 errors or mask real interactions leading to Type 2 errors. •Longitudinal studies are more powerful than cross-sectional ones for inferring genuine within-host interactions, but re-sampling hosts over time can be challenging, with potential bias in follow-up	Cooper et al., 2013; Kreisinger et al., 2014; Lee et al., 2014; Maurice et al., 2015; Morton E. et al., 2015; Šlapeta et al., 2015; Ramanan et al., 2016; Jenkins et al., 2017; Yang et al., 2017; Rosa et al., 2018
Community perturbation experiment (anti-parasite treatment)	•Can inform about how parasite removal affects the gut microbiota •Useful when paired with controlled infection experiments, as parasite addition and removal may have different effects •Parasite removal may be more relevant for understanding the likely impact of disease control measures than controlled infection experiments	•Anti-parasite drugs may have direct effects on microbes as well as indirect effects via parasite removal, complicating interpretation of results •In natural populations, re-sampling animals for longitudinal analysis of treatment effects can be challenging, with potential bias in drop-outs	Cooper et al., 2013; Houlden et al., 2015; Maurice et al., 2015; Yang et al., 2017
Community perturbation experiment (antibiotic treatment)	•Can inform about how depletion of gut microbes affects a phenotype of interest (e.g., parasite colonization, reproduction, or survival, or parasite-mediated effects on host health) •Relevant to understanding the impact of real-world antibiotic treatment	•Current antibiotics are a blunt experimental tool as they are often broad-spectrum, such that pinpointing effects to a particular bacterial group or species is usually impossible	Mansfield and Urban, 1996; Hayes et al., 2010; Reynolds et al., 2014
Co-housing experiments	•Can inform about whether a characterized phenotype is transmissible and due to microbial alterations alone	•Transfer of microbes from one group of interest to another can go in either direction, with no *a priori* expectation about which will occur. Thus, *post-hoc* analysis of which group assimilated the other's microbiota, and the impact of this on phenotypes is necessary	Ramanan et al., 2016

### Enabling study comparability and synthesis

The current literature on parasite-microbiota interactions often describes heterogeneous findings even on the same or similar host-parasite systems. This can result from variation in the host or parasite strain used, experimental design, sampling types and time points, and analysis pipelines. Heterogeneity in a host's baseline microbiota, for example across mouse vendors, can also lead to contrasting immune responses (Ivanov et al., 2008) that may impact infection outcomes, and has even been made use of to help understand microbiota-parasite interactions (Burgess et al., 2014). Using separately housed littermates in experiments may help minimize the influence of potential covariates when analyzing the gut microbiota (Mamantopoulos et al., 2017). An awareness of these methodological sources of variation will be critical to aid progress in this field. Repeating and standardizing study designs as much as possible as well as utilizing common analytical tools will aid cross-study comparison and help reveal true, repeatable parasite-microbiota interactions. For example, studies based on 16S sequencing often report microbiota changes at select bacterial taxonomic levels, which makes comparison across studies difficult. Analytical methods that avoid such choices and assess changes at all taxonomic levels, for example depicting group differences with tree-like visualizations rather than taxonomic bar charts (Segata et al., 2011; Foster et al., 2017) will be a useful tool for the future. Just as large projects like the Human Microbiome Project (Turnbaugh et al., 2007) and Earth Microbiome Project (Gilbert et al., 2014) have attempted to standardize wet lab pipelines as much as possible, and standardized analysis pipelines for 16S data are being published (e.g., Callahan et al., 2016; Comeau et al., 2017), such standardization could aid comparability among studies working at this new interface of parasitology and microbiology. Perhaps more importantly, as researchers will no doubt require some flexibility in their analytical approach to address particular questions, data from published work needs to be made freely available and open access wherever possible, to enable future meta-analytic and synthetic studies that combine multiple datasets to draw out commonalities and understand why differences arise.

### The contribution of studies in natural systems and through an ecological lens

While lab-based studies in model organisms provide a powerful means of understanding parasite-microbiota interactions and their mechanistic basis, work in natural settings is also important, as lab-based studies may not accurately reflect what happens in complex real-world settings where within-host communities are more diverse and dynamic, and hosts experience stronger environmental fluctuations and natural selection. Recent studies have found large differences in both immune traits and the gut microbiota between lab strains of house mice and their wild counterparts, which can impact the outcome of infection (Beura et al., 2016; Abolins et al., 2017; Rosshart et al., 2017). Movement of laboratory mice into outdoor enclosures has further shown that changes to the host environment can rapidly shift host immune responses, gut microbes, and susceptibility to infection (Leung et al., 2018). These natural environments expose hosts to environmental microbes, require them to endure natural challenges such as the need to navigate a complex environment, and provide them with the opportunity to compensate energetically for infection with additional and selective feeding (Budischak et al., 2018). Thus, studies investigating parasite-microbiota interactions under more natural conditions can improve our ability to translate findings from the laboratory to the field, and to understand where lab conditions do, and do not, accurately reflect processes occurring in free-living animals. Such studies need not be limited to purely correlational or cross-sectional studies, and can employ experimental approaches (e.g., perturbation experiments; Table 2), longitudinal sampling, and the use of semi-natural populations to improve causal inference and tractability.

Overall, studies investigating parasite-microbiota interactions in the vertebrate gut are still in their infancy and many outstanding questions remain (Box 2). As this field moves forward, applying an ecological framework and the use of associated statistical tools (e.g., Fenton et al., 2010) may help us move toward an understanding of the processes mediating interactions between gut bacteria and parasites, and thus better predict the impacts on host health. Ecological approaches can also complement the many reductionist experiments (e.g., using gnotobiotic or transgenic mice) needed to reveal molecular mechanisms underlying particular outcomes. We argue that considering ecological alongside molecular mechanisms of interaction can aid in reconciling apparently contradicting findings among experimental studies on different parasite and microbial taxa, just as considering ecology has previously helped make sense of heterogeneous parasite-parasite interactions within hosts (Graham, 2008; Knowles, 2011). Indeed, investigating complex species interactions, drivers of context dependency in those interactions, and resilience of whole ecosystems are core activities of community ecologists. Ecology is therefore crucial to making sense of the complex world within our guts.

Box 2 **Outstanding questions**

1. Which organisms (parasite, microbes, or host) benefit from the observed effects of parasites on the microbiota? Do parasites benefit such that microbiota changes might reflect a manipulation by the parasite, do hosts benefit such that microbiota changes represent a part of the host's defenses, or are the changes simply a non-adaptive by-product of co-infection?
2. To what extent are parasite and microbe behaviors (e.g., secretions of ES molecules) fixed versus plastic (e.g., depending on interactions with the other domain)?
3. Do parasites and gut microbes interact through overlapping resource requirements, for example competing for the same nutrients or cross-feeding?
4. How frequently do parasites (especially helminths) feed directly on microbes such that their ecological relationship is better understood as predator-prey rather than as competitors?
5. Do different stages of parasite infection alter the microbiota differently?
6. Is the impact of parasitic infection on the microbiota similar across all host ages, or is there a “critical window” during development when parasite-microbiota interactions are more influential in terms of host health?
7. To what extent is malnutrition associated with parasite infection a result of parasite-driven changes to the gut microbiota?
8. Which probiotic species can best improve protective host immune responses to parasite infection?
9. Can administration of prebiotics reduce susceptibility to parasite infection? If so, what microbial taxa are altered with prebiotics leading to this effect?

## Author contributions

JL and SK: contributed to the conception of the work, reviewed and analyzed the primary literature, and wrote the paper; AG: revised it critically for important content.

### Conflict of interest statement

The authors declare that the research was conducted in the absence of any commercial or financial relationships that could be construed as a potential conflict of interest.
