# Supplementary material for: Parasite-Microbiota Interactions With the Vertebrate Gut: Synthesis Through an Ecological Lens
Source: Front Microbiol. 2018 May 14;9:843. doi: 10.3389/fmicb.2018.00843 (PMC5960673; doi:10.3389/fmicb.2018.00843)
Supplement: Supplementary file 1 [file Table_1.pdf]

Supplementary Material

Parasite-microbiota interactions with the vertebrate gut: synthesis through an ecological lens

Jacqueline M. Leung[1]*, Andrea L. Graham[1], Sarah C. L. Knowles[2]*

*** Correspondence:** Jacqueline M. Leung: jmyl@princeton.edu
Sarah C. L. Knowles: sknowles@rvc.ac.uk

1 Supplementary Tables

Supplementary Table S1. Summary table of studies investigating helminth-induced alterations to the gut microbiota in vertebrates

Host	Helminth species	Infective dose	Sampling time (days p.i.)	Sample site(s)	Microbiota diversity	Microbiota composition			Reference
						Phylum	Family	Genus	
**Swine**									
Pig	*Trichuris suis*	2x10^4	21	Luminal contents from colon	No change	↑Deferribacteres<br>↑Proteobacteria		↑*Desulvibrio*<br>↑*Mucispirillum*<br>↑*Paraprevotella*<br><br>↓*Asteroleplasma*<br>↓*Blautia*<br>↓*Coprobacillus*<br>↓*Dorea*<br>↓*Eubacterium*<br>↓*Oribacterium*<br>↓*Oscillibacter*<br>↓*Peptococcus*<br>↓*Scwartzia*<br>↓*Selenomonas*<br>↓*Spirochaeta*<br>↓*Sporobacter*<br>↓*Succinivibrio*	Li et al., 2012

Pig	*Trichuris suis*	2x10^4	53	Luminal contents from colon	N/A	↓Fibrobacteres<br>↓Spirochaetes<br>↓Tenericutes<br>↓Gemmatimonadetes		↓*Fibrobacter*<br>↓*Treponema*<br>↓*Spirochaeta*<br>↓*Dorea*<br>↓*Brachyspira*<br>↓*Mycoplasma*<br>↓*Thermotoga*<br>↓*Actinobacillus*<br>↓*Francisella*<br>↓*Erysipelothrix*<br>↓*Ruminococcus*	Wu et al., 2012
Pig	*Ascaris suum*	5000	14	Proximal gut	No change			↑*Cellulosimicrobium*<br>↑*Facklamia*<br>↑*Haemophilus*<br>↑*Succinivibrio*<br>↑*Turicibacter*<br>↑Paraprevotellaceae uc.<br>↑Pasteurellaceae uc.<br>↑Planococcaceae uc.<br>↑Porphyromonadaceae uc.<br>↑Succinivibrionaceae uc.<br><br>↓*Catenibacterium*<br>↓Veillonellaceae uc.	Williams et al., 2017
**Rodent**									
Mouse	*Trichuris muris*	20	14, 28, 42, 49, 56, 63, 70, 77, 84, 91	Feces	Decreased alpha diversity on days 41 and 91		↑Rikenellaceae<br>↑Family F16 from TM7 phylum	↑*Mucispirillum*<br><br>↓*Prevotella*<br>↓*Parabacteroides*	Houlden et al., 2015
Mouse	*Trichuris muris*	20	13, 20, 27, 35	Feces for days 13, 20, 27; Luminal contents from colon on day 35	Decreased alpha, beta, and gamma diversity after day 27	↑Firmicutes<br>↑Proteobacteria<br><br>↓Bacteroidetes	↑Lactobacillaceae<br>↑Rikenellaceae<br><br>↓Desulfovibrionaceae<br>↓Ruminococceae<br>↓Erysipelotrichaceae	↑*Bifidobacterium*<br>↑*Lactobacillus*<br>↑*Alistipes*<br>↑*Odirobacter*<br>↑*Oscillibacter*<br>↑*Butyricicoccus*<br>↑*Parasutterella*<br>↑*Sphingomonas*<br>↑*Halomonas*<br>↑*Mucispirillum*<br><br>↓*Parabacteroides*<br>↓*Barnesiella*<br>↓*Allobaculum*<br>↓*Roseburia*<br>↓*Sporobacter*	Holm et al., 2015

Mouse	*Trichuris muris*	25	21, 35	Feces, SI mucosa	Decreased alpha diversity in WT infected animals, but increased alpha diversity in Nod2-/-		↑Lachnospiraceae<br>↑Clostridiales (order)	↓*Prevotella*<br>↓*Bacteroides*	Ramanan et al., 2016
Mouse	*Trichuris muris*	200	20-22, 26-29	Feces	Decreased alpha diversity in re-wilded mice			In the lab:<br>↑*Alistipes* (reversed in re-wilded mice)<br><br>↓*Ruminococcus*<br>↓*Allobaculum*<br>↓*Barnesiella* (reversed in re-wilded mice)	Leung et al., 2018
Mouse	*Heligmosomoides polygyrus*	200	12, 35	Feces, SI mucosa	N/A		↑Clostridiales (order)	↓*Bacteroides*	Ramanan et al., 2016
Mouse	*Heligmosomoides polygyrus bakeri*	200	6, 14, 28	Luminal contents from ileum, cecum, and colon	N/A	↑g-Proteobacterium	↑Enterobacteriaceae<br>↑Porphyromonadaceae	↑*Lactobacillus*<br>↑*Bacteroides*<br>↑*Prevotella*<br><br>↓*Paenibacillus*	Rausch et al., 2013
Mouse	*Heligmosomoides polygyrus bakeri*	200	28	Duodenum, feces	N/A		↑Enterobacteriaceae	↑*Lactobacillus*	Reynolds et al., 2014
Mouse	*Heligmosomoides polygyrus*	N/A	14	Illeum, cecum tissue	N/A		↑Lactobacillaceae<br>↑Clostridaceae<br>↑Porphyromonadaceae<br><br>↓Erysipelotrichaceae<br>↓Clostridiales (order)		Walk et al., 2010
Mouse	*Heligmosomoides polygyrus bakeri*	200	21	Cecal contents	Not consistent		↑Clostridiales (order)	↑*HA-107 E. coli*	Zaiss et al., 2015
Mouse	*Nippostrongylus brasiliensis*	500	11	SI lumen; feces	No change	↑Bacteroides<br>↑Actinobacteria<br><br>↓Firmicutes	↑Lactobacillaceae<br>↑S24-7 Family<br>↑Coriobacteriaceae<br><br>↓Peptostreptococcaceae<br>↓Clostridiaceae<br>↓Turicibacteraceae		Fricke et al., 2015

Mouse	*Trichinella spiralis*	300-500	12	SI and LI luminal contents	N/A		↑Clostridales (order)<br>↑Ruminococcaceae<br>↑Lachnospiraceae<br>↑Lactobacillaceae<br><br>↓Clostridiceae<br>↓Turicibacteraceae		Osborne et al., 2014
Wild mouse	*Heligmosomoides polygyrus; Syphacia* spp.; *Hymenolepsis* spp.	N/A	N/A	Lumen of stomach, SI, cecum, and distal colon	No change	*H. polygyrus*:<br>↑Firmicutes<br>↓Bacteroidetes<br><br>*Syphacia* spp:<br>↑Bacteroidetes<br>↓Firmicutes	*H. polygyrus* & *Syphacia* spp:<br>↓Lachnospiraceae<br>↓Lactobacillaceae<br>↓Ruminococcaceae<br>↓Acetobacteraceae<br>↓Sphingomonadaceae<br>↓S24-7<br><br>*Hymenolepis* spp:<br>↑S24-7<br>↑Lactobacillaceae		Kreisinger et al., 2015
Wild mouse	*Heligmosomoides polygyrus*	N/A	N/A	Feces	N/A			↑Escherichia<br><br>↓Lachnospiraceae uc.	Maurice et al., 2015
Rat	*Hymenolepis diminuta*	4	62	Luminal contents of cecum	No change		↑Peptostreptococcaceae	↓*Turicibacter*	McKenney et al., 2015
Rat	*Hymenolepis diminuta*	10 each for 3 days	3 consecutive days followed by a 7-10 day gap for 50 days	Feces	No change in alpha diversity; shifts in beta diversity		↑Erysipelotrichaceae<br>↑S24-7<br>↑Ruminococcaceae<br>↑Mollicutes order RF39	↑*Turicibacter*<br>↑*Sutterella*	Parfrey et al., 2017
Hamster	*Opisthorchis viverrini*	50	42	Feces from colon and rectum; bile from bile duct	Increased alpha diversity	↑Spirochaetes	↑Ruminococcaceae<br>↑Lachnospiraceae<br><br>↓Porphyromondaceae<br>↓Erysipelotrichaceae	↑*Lactobacillus*	Plieskett et al., 2013
Rabbit	*Trichostrongylus retortaeformis*	1000 every 7 days	0, 15, 30, 60	Mucosa of duodenum	Decreased alpha diversity	↑Proteobacteria<br>↑Spriochaetes<br><br>↓Firmicutes	↑Leptospiraceae<br>↑Desulfobacteraceae<br><br>↓Ruminococcaceae<br>↓Porphyromonadaceae<br>↓Bacteroidaceae	↑*Leptomna*<br>↑*Desulfocella*<br><br>↓*Ruminococcus*<br>↓*Bacteroides*	Cattadori et al., 2016
**Ruminant**									
Goat	*Haemonchus contortus*	5,000	50	Lumen of abomasum	No change	↓Euryarchaeota	↑Pasteurellales (order)<br>↑Prevotellaceae	↑*Prevotella*<br>↑*Selenomonas ruminantium*	Li et al., 2016

Sheep	*Haemonchus contortus*	5,000	7, 50	Rumen and abomasum fluids	Increased alpha diversity	In abomasum fluids:<br>↑Lentisphaerae*<br>↑Bacteroidetes*<br>↑Tenericutes*<br>↑Candidate_division_TM7*<br>↑Synergistetes<br>↑Firmicutes*<br>↑Elusimicrobia<br>↑Fibrobacteres*<br>↑Proteobacteria*<br>↑Cyanobacteria<br>↑Actinobacteria*<br>↑Spirochaetae<br>*Also ↑ in rumen fluid		↑*Prevotella*	El-Ashram et al., 2017
Calf	*Ostertagia ostertagi*	1x10^5	14	Lumen of abomasum	No change			↑*Ethanoligenens*<br><br>↓*Subdoligranulum*	Li et al., 2011
**Companion animal**									
Dog	*Ancylostoma caninum*	N/A	N/A	Feces	No change				Slapeta et al., 2015
Cat	*Toxocara cati*	N/A	N/A	Feces	No Change	↑Actinobacteria	↑Coriobacteriaceae<br>↑Enterococcoaceae	↑*Collinsella*<br>↑*Enterococcus*<br>↑*Dorea*<br>↑*Ruminococcus*<br><br>↓*Bulleidia*<br>↓*Jeotgalicoccus*	Duarte et al., 2016
**Primate**									
Macaque	*Trichuris trichiura*	1,000	98	Colon mucosa	Increased alpha diversity (although not statistically significant)	↑Tenericutes<br>↑Bacteroidetes<br>↑Elusimicrobia<br><br>↓Cyanobacteria<br>↓ZB2		↓*Streptophyta*	Broadhurst et al., 2012
Human with Celiac's Disease	*Necator americanus*	20	0, 56	Feces	No change				Cantacessi et al., 2014
Human with Celiac's Disease	*Necator americanus*	20	168, 252	Mucosa of duodenum	Increased alpha diversity at 168 days; returned to baseline at 252 days	↑Bacteroidetes			Giacomin et al., 2016

Human	Mix of *Trichuris* spp., *Ascaris* spp., and hookworms	N/A	N/A	Feces	No difference in alpha diversity; Increased beta diversity		↑Verrucomicrobiaceae<br>↑Enterobacteriaceae<br><br>↓Bacteroidaceae<br>↓Leuconostocaceae	↑*Lactococcus*<br>↑*Akkermansia*<br>↑Undefined Enterobacteriaceae<br><br>↓*Bacteroides*	Jenkins et al., 2017
Human	Mix of *Trichuris* spp., *Ascaris* spp., and hookworms	N/A	N/A	Feces	Increased alpha diversity		↑Paraprevotellaceae	↓*Bifidobacterium*	Lee et al., 2014
Human	Mix of *Trichuris trichiura* and *Ascaris lumbricoides*	N/A	N/A	Feces	*T. trichiura* only: No change<br><br>Mix of *T. trichiura* & *A. lumbricoides*: Decreased alpha diversity			Mix of *T. trichiura* and *A. lumbricoides*:<br>↑*Streptococcus*	Cooper et al., 2013
Human	Mix of *Trichuris* spp., *Ascaris* spp. and hookworms	N/A	N/A	Feces	Increased alpha diversity	After self-clearing*:<br>↑Synergistetes<br>(*Note: These are individuals going from worm-positive to worm-negative)	After deworming*:<br>↓Sutterellaceae<br><br>After self-clearing*:<br>↑Porphyromonadaceae<br>↑Bacteroidaceae<br>(*Note: These are individuals going from worm-positive to worm-negative)	After deworming*:<br>↑*Sphingobacterium*<br>↑*Clostridium_XVIII*<br>↓*Xylanibacter*<br>↓*Leuconostoc*<br>↓*Butyricimonas*<br><br>After self-clearing*:<br>↑*Parabacteroides*<br>↑*Bacteroides*<br>↓*Olsenella*<br>(*Note: These are individuals going from worm-positive to worm-negative)	Rosa et al., 2018
Human	*Enterobius vermicularis*	N/A	N/A	Feces	Increased alpha diversity			↑*Alistipes*<br>↑*Faecalibacterium*<br><br>↓*Fusobacterium*<br>↓*Veilonella*<br>↓*Megashpaera*<br>↓*Acidaminococcus*<br>(Note: The corrected p-values (FDRs) for all these taxa were >0.05.)	Yang et al., 2017
